# Epidemiological surveillance: a brief history and the experiences of the United States and the state of São Paulo

**DOI:** 10.1590/S2237-962220220002000028

**Published:** 2022-06-13

**Authors:** Rita Barradas Barata

**Affiliations:** 1 Faculdade de Ciências Médicas da Santa Casa de São Paulo, Departamento de Saúde Coletiva, São Paulo, SP, Brazil

**Keywords:** Epidemiological Monitoring, Disease Outbreaks, Epidemics, History

## Abstract

**Objective:**

The objective of this narrative review was to list some historical aspects of epidemiological surveillance, a technological intervention model initially designed to help control communicable diseases in the last century.

**Methods:**

This narrative was built based on texts selected to record the development of epidemiological surveillance in the United States and in the state of São Paulo, Brazil.

**Results:**

The origins of some of the actions that constitute epidemiological surveillance activities are presented, as well as a brief history of the establishment of the originally named Center for Disease Control, a United States agency that is held up as an example in relation to the way surveillance has been performed, practically all over the world. Likewise, we outline the paths that led to the establishment of the surveillance system in the state of São Paulo, drawing some parallels with the Brazilian system.

**Conclusion:**

The narrative concludes with a conceptual differentiation between epidemiological surveillance, monitoring and health surveillance.

## INTRODUCTION

This is a narrative review of the historical origins of epidemiological surveillance, a technology for control of diseases and health conditions, which was built *pari passu* with the constitution of epidemiology as a scientific discipline with effect from the 19^th^century, through to its consolidation in the 20^th^century. In order to build this narrative, the history of the United States Centers for Disease Control and Prevention (CDC) was used, to a large extent, given the importance that the institution has had in epidemiological surveillance all over the world. Aspects of the establishment and implementation of epidemiological surveillance in the state of São Paulo, Brazil, and how much this experience influenced the establishment of the Brazilian national epidemiological surveillance system have been included.

## BACKGROUND

Probably the first component of epidemiological surveillance used to contain contagion was the surveillance of contacts in Venice in the 14^th^century, with the imposition of quarantine on ships arriving from the East, with crew members affected by cholera, smallpox or plague.^1^

Quarantine and sanitary cordons were measures to control the spread of diseases, proposed in the context of the contagion theory, and health surveillance consisted of monitoring sick people’s contacts until they were considered free from risk of disease. free.^2^

Lack of knowledge of the exact amount of time needed for case observation gave rise to quarantine, established arbitrarily as a safety period prior to contacts being authorized to move freely once more.^2^

Monitoring of deaths and cases during outbreaks and epidemics was only routinely implemented in the United Kingdom during the 19^th^century, under the command of William Farr, a British epidemiologist and statistician. The objective of comparing patterns of mortality in different years was merely to identify the emergence of epidemics. Case and death investigations were limited to outbreaks and epidemics, but there was no systematic practice of active surveillance.^3 ,4^

**Table:** 

Study contributions	
**Main results**	Narrative review presenting the origins of epidemiological surveillance, a brief history of the establishment of the United States system and surveillance system in the state of São Paulo. The article also brings concepts of epidemiological surveillance, monitoring and health surveillance.
**Implications for services**	The origins and development of epidemiological surveillance can encourage professionals, increase their commitment and highlight epidemiological objectives, surpassing the bureaucratic character of data recording.
**Perspectives**	The need for professionals who have epidemiology quaifications is essential for good epidemiological surveillance practices, as well as the stability of a technical staff for good performance of this activity in health services.

Until then, compulsory notification, which had been established by royal edict in the 17^th^century, was only complied with in periods of unusual increase in the number of cases.^3^

With the development of microbiology, there was great progress in research on potential etiological agents and the transmission process, with the elaboration of essential concepts for further establishment of epidemiological surveillance.^5^ These concepts form the basis of the process called the ‘transmission chain’, namely: source of infection, carriers, modes of transmission, vectors and vehicles of contamination, reservoirs, definitive and intermediate hosts, as well as prophylaxis measures to interrupt the process.^5 , 6^

The steps of the process were also investigated, enabling the establishment of different times and intervals, important for understanding the dynamics of the infection process at the population level: latency period, incubation period, transmission period, convalescence.^5 , 6^

The conditions necessary for the formulation of epidemiological surveillance as it is currently conceived of only emerged in the 20^th^century. On the eve of World War II, in 1939, the United Kingdom created a public health laboratory network aimed at identifying infectious disease outbreaks and episodes of chemical poisoning, adopting the idea of sentinel services and the concept of epidemic intelligence, similar to military intelligence ( Box 1 ).^1^

**Box 1:** International and São Paulo state, Brazil, milestones in the development of epidemiological surveillance

Period	Important facts in the 20^th^century
1939	Establishment of the public health laboratory network and interrupted. The prestige intelligence in the United Kingdom.
1942	Establishment of Malaria Control in War Areas (MCWA), CDC’s predecessor, in the United States.
1946	Establishment of Communicable Disease Center (CDC) to replace MCWA.
1956	Approval of the Malaria Eradication Campaign by the General Assembly of the World Health Organization (WHO). Epidemiological surveillance is one of the stages of the eradication program.
1960	Association between epidemiological surveillance and national immunization programs in the United States.
1966	Incorporation of the sexually transmitted diseases program into the CDC.
1967	Investigation of possible smallpox outbreak among Bolivian troops trying to capture the guerrillas commanded by Che Guevara.
1970	Agency’s name changed to Center for Disease Control, following the expansion of the scope of actions for chronic diseases, smoking, nutritional, environmental and accident problems.
1972	Tuskegee scandal, which the CDC inherited with the incorporation of the sexually transmitted disease program.
1976	Swine flu episode fiasco.
1980	Serious crisis faced by CDC under the Ronald Reagan administration.
1981	Investigation of the first cases of AIDS.
1990	The agency regained its prestige and it would be put at risk, once again, under Donald Trump’s administration.
	**Important facts in São Paulo**
1930	Special service for combating yellow fever responsible for mass vaccination.
1931	State Department of Education and Public Health, and the Department of Public Health in charge of the coordination of the Butantã, Pasteur and Bacteriológico Institutes, Hospital de Isolamento and Inspetoria Geral de Higiene.
1947	Establishment of the Department of Public Health Services and Social Assistance.
1967	Smallpox eradication campaign based on mass vaccination and epidemiological surveillance.
1968	Establishment of the Community Health Coordination, including epidemiological surveillance activities – the state’s first vaccination norm.
1974	Negotiations for the purchase of meningococcal vaccines.
1975	Mass vaccination campaign against meningococcal disease.
1975	Establishment of the Health Information Center (CIS) in charge of epidemiological surveillance in the state. Establishment of the National Epidemiological Surveillance System (Law No. 6,259).
1983	State Program for STD/AIDS
1985	CIS became the Epidemiological Surveillance Center (CVE).
1990	Establishment of the STD/AIDS Reference and Training Center and National Epidemiology Center (CENEPI).
2001	Establishment of the Coordination for Disease Control (CCD), bringing together the CVE, Health Surveillance Center (CVS) and Superintendence for Epidemic Control (Sucem).
2003	At the federal level, the establishment of the Health Surveillance Secretariat of the Brazilian Ministry of Health (SVS/MS).

On the other side of the Atlantic Ocean, the United States created Malaria Control in War Areas (MCWA) in the city of Atlanta, Georgia, an initiative that during the wartime, between 1942 and 1946, operated in 13 Southeastern states of the United States where malaria was prevalent. Since antiquity, malaria has been one of the main enemies of any army at war and, therefore, a major strategic concern.

For the United States and part of the British and Australian armed forces, protagonists of the global conflict in the Pacific Ocean, the risk of malaria contagion posed a major challenge. Quinine production areas being dominated by the Japanese made the problem even worse for what were referred to as the Allied Forces. The fact that the British obtained, through espionage in the battles for North Africa, the formula for synthetic antimalarials (chloroquine) developed by the Germans, allowed the Allies to dispense with quinine plantations for the treatment of their troops.^7 , 8^

After the end of World War II, the MCWA was transformed into the Communicable Disease Center (CDC), initially aimed at combatting vector-borne diseases.^7 , 8^

## CDC’s MISSION

The first tasks undertaken by the new United States public health organization were the campaigns to eradicate malaria, endemic typhus and dengue. The return of troops from war areas was faced with apprehension by public health authorities, given the risk of worsening the situation in the country, where there were vectors but not an abundance of sources of infection, enabling them to keep occurrences under control.^7 , 9^

The malaria eradication program consisted of indoor residual spraying with DDT (original acronym for dichloro-diphenyl-trichloroethane), in rural households, drainage services, elimination of Anopheles breeding sites using chemicals and aerial spraying of insecticides in forests. After six years of intensive work, it was possible to replace these actions with epidemiological surveillance activities, that is, localized interventions, triggered by the notification of confirmed cases of the disease.^10^

Based on the United States experience, in 1956, the World Health Organization (WHO) proposed that its member states should support a global campaign for the eradication of malaria, based on the existence of effective instruments to combat vectors, diagnose and treat infection sources, enabling a massive invention to be designed. The argument presented to convince the countries to adhere to the campaign was vector resistance to insecticides and Plasmodium resistance to available treatment, a fact that required a rapid response, among all committed countries, before the control instruments could become ineffective.^11^

This global eradication campaign was designed to be developed in four phases: preparatory; attack; consolidation; maintenance. In the preparatory phase, each country was to identify its endemic areas and take charge of operational preparations for the subsequent phases.

The attack phase consisted of massive use of sprays with DDT in rural households, active tracing and treatment of transmission sources. After reducing incidence, the consolidation phase would begin, focused on the elimination of pockets that were resistant to attack activities.

Finally, the maintenance phase had the objective of establishing an epidemiological surveillance system, aiming at early diagnosis of imported or introduced cases, quickly interrupting the transmission process.^12^

Due to the success achieved in the control of endemic diseases, engineers and entomologists gave space to epidemiologists and public health laboratories, so that epidemiological surveillance technology could be used to control other communicable diseases, instead of being restricted to coping with vector-borne diseases.

In the 1950s, polio epidemics gave the CDC the opportunity to extend its action beyond endemic diseases. The CDC’s chief of epidemiology, Alexander Langmuir, gave the name Epidemic Intelligence Service (EIS) to the activities aimed at detecting and investigating outbreaks and epidemics.^4^ This epidemiological intelligence service began to coordinate field epidemiology training programs, training researchers to work in the State Departments of Health in the United States and in international missions.^7^

In 1955, with the investigation of the polio outbreak that followed failures in the production of some batches of the Salk vaccine, in addition to the emergence of the Asian influenza pandemic in 1957, the risk of extinction faced by CDC and its merger with the National Institutes of Health (NIH) was removed. Director Justin Andrews justified the need to keep the agency in order to monitor the emergence of new diseases, produce information for disease control, develop new methods and work in international health.^1 , 7 , 13^

In the 1960s, epidemiological surveillance was linked to the United States National Immunization Program. Epidemiological surveillance activities in that country, at the state level, consisted of the investigation of all reported cases, identification of sources of contagion and vaccination blockade or other prophylactic measures, while at the federal level, they consisted of the analysis and monitoring of the epidemiological profile, field investigator training and support to state teams, in addition to the investigation of new diseases.

In 1962, President John Kennedy created the national polio immunization program. When this program was created, it included only the Salk vaccine and the triple bacterial vaccine. In 1965, however, it included the measles vaccine as well.^7^

In 1966, once its epidemiological surveillance work was consolidated, the CDC took charge of the sexually transmitted diseases program. At the international level, that same year, President Lyndon Johnson (United States) and Prime Minister Nikita Khrushchev (Soviet Union) proposed that WHO should conduct a global campaign for the eradication of smallpox, based on mass vaccination for the world population and epidemiological surveillance actions.^7 , 14^

In 1967, CDC teams investigated a possible smallpox outbreak among Bolivian troops engaged in the capture of guerrillas commanded by Che Guevara. That was arenavirus – not smallpox – and this would be just one of many episodes in which the CDC’s actions would be involved with the activities of the United States intelligence agencies.^7^

With regard to the 1960s, the importance of epidemiological surveillance was confirmed during the Hong Kong flu pandemic, which affected 53 million of people from United States causing 20,000 deaths, and the immunization campaign against rubella, after the confirmation that it had a potential to cause severe congenital malformations, in addition to a large number of cases of deafness.^7^

## EXPANSION OF CDC’s MISSION

In the 1970s, the agency’s name changed to Center for Disease Control, establishing the expansion of the agency’s mission, which began to incorporate responsibility for health statistics, monitoring of chronic diseases, nutritional problems, tobacco control, environmental issues, nuclear accidents, bioterrorism and emerging diseases.^7^

In 1972, the agency suffered huge damage by the revelations of the Tuskegee scandal. That was a study that had begun in 1936, developed by the sexually transmitted diseases (STD) program of the United States Public Health Service (PHS), involving a Black population comprised of men with syphilis in the state of Alabama. In fact, the study consisted of not treating people in order to observe the natural evolution of syphilis without therapeutic intervention, although antibiotic treatment had been available to the population since the 1940s. As a result of this experiment, several women and children were infected by syphilis during follow-up and in 1972, there were only 76 participants alive who were taking part in the study. Although it had not been an initiative of the CDC, the Tuskegee study was not interrupted when the STD/PHS division was incorporated by the agency in 1966.^7^

The loss of prestige caused by this scandal, involving unethical behavior and structural racism, worsened after the failure to address the flu epidemic in 1976. Moreover, the CDC predicted a calamity situation similar to that of the Spanish flu in 1917-1918, when the first cases of H1N1 flu (swine flu) were reported in 2009-2010. A large amount of resource had been mobilized for vaccine production and holding a national campaign. However, there was no explosion of occurrences, as announced and, in addition, many cases of Guillain-Barré syndrome were reported after vaccination. The campaign had to be interrupted. The prestige of the agency was greatly affected and its credibility was even more damaged with the investigation of the disease known as “legionnaires’ disease”, as identification of the etiological agent proved to be quite difficult and time-consuming.^7^

In the 1980s, under the Ronald Reagan administration, the CDC was in a very difficult situation as it was the target of successive budget cuts and, mainly, discredited in its technical competencies. It was precisely in this context that the first cases of the acquired immunodeficiency syndrome (AIDS) epidemic emerged, which would mobilize all the technical and scientific resources of the agency. After the beginning of the investigations on the unprecedented event, it was possible to identify the transmission characteristics of AIDS and classify it as a sexually transmitted disease. Further investigations also showed that the infectious agent, possibly a virus, could be transmitted through contaminated blood and blood products, sharing syringes, and via transplacental route. About three years after the emergence of the disease, its etiological agent was identified, diagnostic capacity and development of increasingly effective treatment were expanded. The CDC and the epidemiological surveillance system played an important role in this process, despite the unfavorable economic and political conditions experienced in the 1980s.^7^

The agency regained its national and international relevance in the following years, when it devoted itself to the elucidation of emerging diseases and the investigation of internal and external episodes of bioterrorism,^7^ in addition to incorporating other activities that led, once again, to its name being changed, so that it came to be called Centers for Disease Control and Prevention, although the acronym CDC remained the same.

## EPIDEMIOLOGICAL SURVEILLANCE IN THE STATE OF SÃO PAULO: A BRIEF HISTORY

The Inspetoria Geral de Higiene ( Inspector General of Hygiene) of São Paulo state was created in 1891, with the objective of coordinating environmental sanitation, supervising professional practice and controlling communicable disease epidemics. The state Health Code, developed with the contribution of engineer Theodoro Sampaio, and approved in 1894, established compulsory notification of diseases that required hospital isolation and disinfection of homes: plague, yellow fever, cholera, smallpox, scarlet fever, measles, diphtheria and pertussis.^15^

1880, the Hospital de Isolamento, now called Instituto Emílio Ribas, was built for people with smallpox, then expanded in 1896 for isolation of other diseases. In 1892, the Instituto Bacteriológico, now called Instituto Adolfo Lutz, was created for the diagnosis of epidemic and endemic diseases. In 1899, the Instituto Serumtherápico was founded, now called Instituto Butantã, for serum and vaccine production. Finally, in 1903, the Instituto Pasteur was created in order to coordinate the control of human and animal rabies.^15^

Control actions were targeted exclusively at epidemic diseases and rural endemic diseases, which posed a serious threat to health and safety in cities, and to agricultural production in the countryside.

In the first three decades of the 20^th^century, four groups of diseases predominated in the epidemiological profile of the state: vector-borne diseases (urban yellow fever, bubonic plague, malaria, tegumentary leishmaniasis, Chagas disease); parasitic diseases (schistosomiasis and hookworm); waterborne diseases (typhoid fever and other types of diarrhea); and airborne diseases (tuberculosis, smallpox, meningococcal disease, scarlet fever and diphtheria).^16^

Spanish flu was the biggest pandemic at the beginning of the last century, accounting for 117,000 cases and 5,331 deaths in the state capital alone, in a short period of time, between six and eight weeks.^17^

The initiatives of the Inspetoria Geral de Higiene included i) sanitary engineering work aimed to transform the environment and reduce the generation of diseases, and ii) the organization of specific programs for each of the diseases in question. These programs, called ‘vertical programs’, established services and actions aimed at specific problems.^15 , 16^

In 1930, the Special Service to Combat Yellow Fever, in charge of mass vaccination, was created. In 1931, the State Department of Education and Public Health was established, when the Department of Public Health began to be in charge of the health service and coordination of the Institutes. The Department was reformulated in 1947, becoming the Department of Public Health Services and Social Assistance, therefore separated from the Department of Education.^15^

In the period between 1930 and 1964, there was a great change in the epidemiological profile of the São Paulo state due to the impact of urbanization, with several polio outbreaks and increased incidence of measles, for which there was no vaccine, while agglomeration facilitated transmission. Polio vaccination campaigns were conducted with Sabin vaccine, and the Executive Immunization Group was created, in charge of the development of the first immunization schedule, for 1964-1965.^15 , 16^

During more than 30 years, the system of compulsory notification of communicable diseases with epidemic potential served as a system for notification of suspected and confirmed cases, without case investigation, contact tracing or adoption of prophylactic measures, except during outbreaks.

Smallpox Eradication Campaign, initiated in 1967 with mass vaccination for 90% of the population in the state of São Paulo, intensified epidemiological surveillance of suspected cases and tracing of individuals that had not been immunized yet. Such procedures remained in force until the disease was declared eradicated in 1975.^15^

In 1968, State Health Secretary Professor Walter Sidney Pereira Leser promoted the reorganization of the State Health Department (SES/SP), structuring several administrative sectors in charge of the management of primary health care centers, institutes and technical services, hospital care and care for individuals with mental illness. Vertical programs were incorporated into the existing structures and implemented by health centers. Epidemiological surveillance began to take place under the coordination of the regional health boards, with teams of sanitarians who had taken a specialization course at the Faculdade de Saúde Pública do Estado de São Paulo (FSP/USP). Endemic disease control fell under the responsibility of the Superintendência de Saneamento Ambiental, later called Superintendência de Controle de Endemias (SUCEN), bringing together engineers, entomologists and sanitarians.^15^

In 1968, the state’s first vaccination norm was defined, including oral BCG vaccine (Bacillus Calmette-Guerin), triple bacterial vaccine (DTP: diphtheria; tetanus, pertussis), polio, measles and smallpox vaccines. In 1969, the Health Department created the career of Public Health Physician and, through an agreement with FSP/USP, specialization courses were offered, in order to increase the number of graduates and fill the career positions.^15^

In 1970, the biggest meningococcal disease epidemic recorded in the country and probably in the world, began, extending until 1977. The usual incidence rate of the disease in the city of São Paulo was 1.90 case per 100,000 inhab. In the first year of the epidemic, it was 2.30 cases, reaching 169.10/100,000 inhab. at the peak of the epidemic in 1974. In 1975, after mass vaccination, the incidence rate of the disease decreased to 48.30 cases/100,000 inhab., returning to the endemic level as of April 1977.^18^

In 1974, Professor Walter Leser returned to SES/SP as its Secretary and led efforts with federal authorities to develop actions aimed to address the epidemic, establishing a hospital care network for sick people and the coordination of a mass vaccination campaign. There were many uncertainties regarding the impact of the campaign, given that there was little previous experience with the use of the vaccine in epidemic contexts, insufficient number of doses of vaccine on the market for Brazilian demand and lack of conjugate meningitis A and meningitis C vaccines. The immunity provided would probably be of short duration, and there were a large number of people exposed to the disease. In the second half of 1974, given the chaos observed in patient care, negotiations began with the French Mérieux Institute of France for the production of a conjugate vaccine in a sufficient quantity to meet the Brazilian demand, estimated at 60 million doses.^18^

The campaign was set for the end of April, in the city of São Paulo, planning to vaccinate the entire population aged 6 months and over in just four days. A total of 280 vaccination sites and mobile teams were organized to work in areas with the highest population density. A total of 1,329 vaccinators were recruited throughout the country, 295,000 posters were distributed encouraging the population to get vaccinated, and a military operation was carried out in order to ensure the distribution of supplies and *PedO-Jets to be used in vaccine administration* . In four days, 11 million people living in the metropolitan region of São Paulo were vaccinated. There was a significant decrease in incidence as early as May, continuing to fall up until the extinction of the epidemic two years later, in April 1977.^18^

As a consequence of the lessons learned during the meningococcal disease epidemic, SES/SP, under the direction of Professor Chester Luiz Galvão Cesar, the Health Information Center (CIS) was established. The CIS was responsible for coordinating epidemiological surveillance activities, reviewing procedures, drafting and publishing the surveillance manual and designing the system instruments.^15^

The National Epidemiological Surveillance System was created in 1975, with the publication of Law No. 6,259/1975, which established a system similar to that of São Paulo for the whole country. The following year, Decree No. 78,231/1976 regulated the law and developed epidemiological surveillance actions, as well as the National Immunization Program (PNI) and compulsory notification rules. This framework was developed and implemented under the coordination of Professor Edmundo Juarez in charge of the Epidemiological Surveillance Secretariat, and Professor José Carlos Seixas, Executive Secretary, both working for the Ministry of Health.^15^

With regard to São Paulo, the first government of the state elected by universal suffrage, after the civil-military dictatorship, put Professor João Yunes in charge of SES/SP. In 1983, his first year as a Secretary of State for Health, under pressure from public opinion and groups concerned about the growth of AIDS cases in the United States, the São Paulo State STD/AIDS Program was launched. Two years later, in 1985, the CIS became the Epidemiological Surveillance Center and it was managed by Professor Alexandre Vranjac.^15^

A new restructuring of SES/SP, during the Orestes Quércia government, put an end to the public career of Public Health Physician in the state, and the former Regional Health Boards were replaced by the Regional Health Offices, which were more focused on social care and less dedicated to public health actions. Epidemiological surveillance began to be performed by employees without any training in epidemiology or public health, and gradually it was transformed into a simple data recording system. There were exceptions, especially in regions that had medical schools and departments of social medicine or public health, which supported the actions of municipal and regional health departments.^15^

In the 1990s, the STD/AIDS Reference and Training Center was created. It was in charge of prevention, diagnosis and treatment of human immunodeficiency virus (HIV) and AIDS in the state. Given the relevance and complexity of coping with the HIV/AIDS epidemic, a vertical program was once again developed, separating the disease-related activities from the epidemiological surveillance structures in force.^15^

The beginning of the decade also witnessed the creation of National Epidemiology Center (CENEPI) by the Ministry of Health, in order to give visibility to epidemiology actions not only in the context of epidemiological surveillance, but also in the analysis of the health situation and evaluation of policies and action programs.

With the arrival of the 21^st^century, CENEPI was replaced by the Health Surveillance Secretariat of the Brazilian Ministry of Health (SVS/MS), strengthening epidemiological actions and activities at the national level. In São Paulo, two important facts marked this period: the creation of the Coordination for Disease Control (CCD), managed by Luiz Jacintho da Silva, and the creation of the São Paulo State Training Program in Epidemiology Applied to the Services of the Brazilian National Health System, aimed at the formation of field epidemiologist teams in order to support the actions of the current Regional Health Departments (DRS/SP) regarding the investigation of outbreaks and epidemics, like the programs that the CDC of the United States has developed since the 1960s.^15^

In the first two decades of this century, several challenges have reinforced the need for an active, technically competent, timely and effective epidemiological surveillance system. The H1N1 influenza pandemic from 2009-2010, the yellow fever epidemic in Botucatu/SP region in 2009, the measles epidemics in 1997 and 2019, the yellow fever epidemic in the Metropolitan Region of São Paulo, in 2018, and finally, the COVID-19 pandemic in 2020, were unusual episodes, which demonstrate how far away is the possibility of a world where communicable diseases will no longer pose a threat to the human population.

Epidemiological surveillance, as an appropriate technology for the control of communicable diseases, has become increasingly relevant and indispensable. However, it is not only an information system for health actions, but also a system capable of capable of early identificaton of new challenges and timely intervention, thus interrupting transmission. Therefore, the necessary resources and technologies need to be available and, above all, qualified professionals who are aware of the task assigned to them.

In order to conclude this narrative review on the institution of the epidemiological surveillance system, it is necessary to differentiate three concepts that are often unclear: public health surveillance; health situation monitoring; and epidemiological surveillance.

Public health surveillance has been defined in Brazil as systematic and continuous collection, analysis and interpretation of health data in order to plan, implement and evaluate public health practices. It is confused with epidemiological practice in health services, extending to environmental interventions and the evaluation of technologies.^19 , 20^

On the other hand, health situation monitoring consists of monitoring and permanent analysis of the epidemiological profile, aiming to detect changes in health status, in the environment or in the distribution of risk factors in order to guide the development of policies and programs.^21^

Finally, epidemiological surveillance comprises the set of actions triggered at the local level, after the identification of a suspected case of a disease or event for which notification is compulsory, or other health problems to which this technology can be applied, aiming to prevent the emergence of new cases or events. Epidemiological surveillance is usually part of broader control programs, performing very specific functions.^21^
