# ERRATUM

**DOI:** 10.1590/S2237-96222023000100031

**Published:** 2023-03-24

In the article **“Epidemiological surveillance: a brief history and the experiences of the United States and the state of São Paulo”**, doi: 10.1590/S2237-96222022000200007, published on Epidemiology and Health Services, 31(2)e2021115, 2022, in the page 1:

Original text:

10.1590/S2237-96222022000200007

Corrected text:

10.1590/S2237-962220220002000028

